# Early outcomes of South African children with persistent viral non-suppression following switch to dolutegravir-based antiretroviral therapy

**DOI:** 10.4102/sajhivmed.v27i1.1842

**Published:** 2026-08-26

**Authors:** Mila Guerrini, Minette Maré, Claire Hulsman, Leonore Greybe, Juanita Lishman, Clair Edson, Thania Hisham, Lisa Frigati, Helena Rabie

**Affiliations:** 1 Department of Paediatrics and Child Health, Faculty of Medicine and Health Sciences, Stellenbosch University, Cape Town, South Africa; 2 Department of Paediatrics and Child Health, Faculty of Medicine and Health Sciences, Tygerberg Hospital, Cape Town, South Africa; 3 Desmond Tutu TB Centre, Faculty of Medicine and Health Sciences, Stellenbosch University, Cape Town, South Africa; 4 Family Centre for Research with Ubuntu, Department of Paediatrics and Child Heath, Faculty of Medicine and Health Sciences, Stellenbosch University, Cape Town, South Africa

**Keywords:** HIV, dolutegravir, antiretroviral therapy, children, persistent viral non-suppression, virological failure

## Abstract

**Background:**

Since 2019 dolutegravir-based antiretroviral therapy (ART) has been introduced for South African children and adolescents living with HIV (CALHIV), including those with persistent viral non-suppression. Current guidelines recommend the reuse of abacavir when switching CALHIV weighing < 30 kg with persistent viral non-suppression to dolutegravir-based ART.

**Objectives:**

We aimed to describe the viral suppression rates, clinical outcomes and risk factors for non-suppression in CALHIV switching to dolutegravir-based ART following persistent viral non-suppression.

**Method:**

We performed a retrospective cohort study at Tygerberg Hospital in Cape Town, South Africa.

**Results:**

We identified 39 children, 18 girls (46.2%). Median age at switch to a dolutegravir-based regimen was 7.2 years. Median duration on treatment pre-switch was 5.6 years. After switching to a dolutegravir-based regimen, 21 children (55.3%) achieved viral suppression. Abacavir was recycled with dolutegravir in 31 children (available viral load for 30/31) of whom 50.0% suppressed. Treatment interruptions were common, with 26 (66.7%) and 15 (38.5%) children experiencing at least one interruption in care before and after switching to a dolutegravir-based regimen, respectively.

**Conclusion:**

We provide evidence that recycling abacavir is safe in children with persistent viral non-suppression switching to dolutegravir. Supporting adherence and preventing therapy interruption is key to success.

**What this study adds:** This real-world study provides programmatic data demonstrating that switching children with persistent viral non-suppression to dolutegravir-based ART achieves a reasonable rate of viral suppression.

## Introduction

Children and adolescents living with HIV (CALHIV) continue to have lower viral suppression rates than adults.^1,2^ In South Africa, there are approximately 150 000 CALHIV and the viral suppression rate in those with available data is approximately 50%.^3,4^ Children aged 1–4 years old have the lowest rate of viral suppression, at 36.3%.^4^ Delayed access to appropriate drugs and fixed-dose combinations (FDC) for children and adolescents across all age groups contribute to this poor suppression rate.^5^

Dolutegravir, a preferred integrase strand transfer inhibitor with non-inferior or superior efficacy when compared to efavirenz, raltegravir, and boosted protease inhibitor-based regimens, was approved for use in adults in 2013.^6,7^ However, paediatric-appropriate formulations of dolutegravir were only licensed by the Food and Drug Administration in 2020 based on data from the IMPAACT P1093 and ODYSSEY studies.^8,9,10,11,12,13,14,15^ The ODYSSEY and CHAPAS-4 studies, along with routine data, confirm the efficacy of dolutegravir in CALHIV with persistent viral non-suppression following a switch to dolutegravir.^12,16,17^

In line with the 2018 WHO guidelines, the 2019 South African guidelines recommended dolutegravir as the preferred initial regimen, as well as for switching in individuals with persistent viral non-suppression.^18,19,20,21^ However, the 10 mg scored dispersible formulation, appropriate for use in children weighing < 20 kg, only became available in 2022.^22^

Switching to dolutegravir in the presence of persistent viral replication, as well as switching during childhood or adolescence, are risk factors for developing dolutegravir resistance.^23^

In adults with persistent viral replication switching to dolutegravir, maintaining a backbone of tenofovir disoproxil fumarate (TDF) with lamivudine or emtricitabine is superior to switching to zidovudine with lamivudine,^24,25,26,27,28^ however, adult doses of TDF can only be used in children aged ≥ 10 years and weighing ≥ 30 kg.^21^ In the CHAPAS-4 study, children with persistent viral replication randomised to tenofovir alafenamide fumarate (TAF) and lamivudine with dolutegravir were more likely to achieve viral suppression than those receiving abacavir or zidovudine and lamivudine with dolutegravir.^16^ Access to TAF remains limited and dosing has not been established across all weight bands.

In South African CALHIV with persistent viral non-suppression weighing < 30 kg, abacavir is recycled.^21^ It remains unclear whether abacavir recycling has the same efficacy as TDF or TAF when switching to dolutegravir in second-line therapy in the programmatic setting.

We aimed to study the outcomes of CALHIV attending Tygerberg Hospital in South Africa who were switched to dolutegravir despite persistent viral non-suppression, and to identify risk factors associated with further persistent non-suppression in this vulnerable cohort.

## Research methods and design

We performed a retrospective folder review of CALHIV aged < 18 years managed at Tygerberg Hospital Paediatric Infectious Diseases Clinic. Eligible participants were CALHIV with persistent viral non-suppression who switched to a dolutegravir-based regimen between 01 January 2021 and 30 June 2024. Persistent viral non-suppression was defined as at least one episode of two or more viral loads ≥ 1000 copies/mL measured at least 3 months apart, and a viral load of > 50 copies/mL at the time of switch to dolutegravir. Children who interrupted therapy prior to the switch to dolutegravir were also included.

As hospitalised CALHIV who initiated dolutegravir as inpatients may have been referred to antiretroviral therapy (ART) facilities closest to their home, we included only those with at least one follow-up visit after switching to dolutegravir.

Children were identified using the clinic database. Clinical data and prior ART history were recorded from paper-based and electronic medical records. Laboratory data including CD4+ T-lymphocyte cell counts and percentages, viral loads and results of drug resistance tests were obtained from the National Health Laboratory Service.

We collected data retrospective data up to 31 October 2024. Data were originally collected up until 31 August 2024; however, because of a national cybersecurity incident in 2024 that disrupted routine follow-up testing, viral load data collection was extended by 2 months to capture delayed or missed measurements, with approval from the Health Research Ethics Committee.

Baseline viral load was defined as the viral load measurement closest to the date of switching to dolutegravir, within the preceding 6 months. Children without this result were not assigned a baseline viral load value. Baseline CD4+ T-lymphocyte count and percentage were defined as the measurement closest to switching, within 6 months before or 1 month after the switch. Viral suppression in children on dolutegravir was defined as a viral load ≤ 50 copies/mL at the last recorded measurement before 31 October 2024, at the date of transfer out, or at the date of last clinical encounter in cases of treatment interruption. Where possible, we categorised the interruption interval as less than 1 month, 1–3 months, and more than 3 months. Time on dolutegravir was calculated from the date of switch to dolutegravir to 31 October 2024, transfer out, or the date of last clinical encounter in cases of treatment interruption.

Weight-for-age, height-for-age, and body mass index (BMI)-for-age *Z*-scores were reported at the time of switch and at the last clinic visit up to 31 August 2024. The *Z*-scores were calculated using the WHO child growth standards and AnthroCalc version 3.0.2 mobile application for children aged less than 5 years and AnthroPlus for older children.^29,30^

Cumulative resistance profiles were analysed using the Stanford HIV Drug Resistance Database and composite resistance scores were reported.^31^

Data were analysed using Statistical Package for Social Sciences (SPSS) version 29 (IBM Corp., Armonk, New York, United States). For continuous variables, we report measures of central tendency (mean, median, mode) and dispersion (standard deviation, range and interquartile range [IQR]) based on the distribution of the data. Categorical variables were summarised using frequencies and percentages. Univariate analyses were performed using Chi-Square tests and univariate binary logistic regression to screen risk factors that might be related to our outcome variable. Variables identified in univariate analyses were entered into a multivariable binary logistic regression model to identify independent risk factors for our outcome variable. The results are reported as odds ratios (OR) with 95% confidence intervals (CI) to quantify the strength of the associations.

### Ethical considerations

The study and waiver of individual consent was approved by the Stellenbosch University Health Research Ethics Committee on 01 August 2024 (project ID: 31025; HREC reference number: U24/06/333). A waiver of informed consent was obtained as the study utilised retrospectively collected routine clinical data from secondary data sources. Any interventions required were performed by the clinician responsible for the child’s care. The study posed minimal risk to participants. Confidentiality was maintained using unique study numbers.

## Results

At the time of screening, 218 patients attended the clinic. Of these, 39 children were eligible for the study, of whom 18 (46.2%) were girls. The median age at ART initiation was 1 year (interquartile range [IQR] 0.18; 1.83) (Table 1). Children were switched to a dolutegravir-based regimen at a median age of 7.2 years (IQR 3.02; 9.73). The rate of viral suppression to ≤ 50 copies/ml was 55.3% (Table 2).

**TABLE 1:** Clinical characteristics and treatment history of children with persistent viraemia switching to dolutegravir (*N* = 39).

Characteristic	*n*	%	Median	IQR
**Sex**				
Female	18	46.2	-	-
**Age at treatment initiation (years) (*N* = 39)**	-	-	0.95	0.18; 1.83
< 1	20	51.3	-	-
≥ 1 to < 5	17	43.6	-	-
≥ 5 to < 18	2	5.1	-	-
**CD4+ T-lymphocyte percent and count at treatment initiation (*N* = 30)**				
CD4+ T-lymphocyte percent	-	-	17.98	12.90; 27.04
CD4+ T-lymphocyte count	-	-	892	477; 1303
**Duration on treatment prior to switch (years) (*N* = 39)**	-	-	5.61	1.77; 8.14
< 2 years	12	30.8	-	-
≥ 2 to < 4 years	4	10.3	-	-
≥ 4 years	23	59.0	-	-
**Combined antiretroviral exposure prior to dolutegravir (*N* = 39)**				
ABC/3TC/LPV/r	20	51.3	-	-
ABC/3TC/LPV/r/ (AZT or D4T)	5	12.8	-	-
ABC/3TC/LPV/r/ (AZT or D4T)/ (EFV or NVP)	7	17.9	-	-
ABC/3TC/ (EFV or NVP	1	2.6	-	-
ABC/3TC/ (LPV and ATV) /(AZT or D4T)	5	12.8	-	-
ABC/3TC/LPV/r/ (AZT or D4T)/ (EFV or NVP)/DRV/r	1	2.6	-	-
**Cumulative exposure to individual ART (years) (*N* = 39)**				
ABC	39	100.0	3.02	1.14; 6.53
3TC	39	100.0	3.69	1.55; 6.90
LPV/r	38	97.4	3.09	1.08; 6.93
ATV/r	5	12.8	1.47	1.09; 1.93
AZT	12	30.7	0.17	0.07; 1.48
d4T	8	20.5	3.46	2.67; 4.37
EFV	2	5.1	4.32	3.41; 5.23
NVP	7	17.9	0.08	0.06; 0.17
DRV/r	1	2.6	1.38	1.38; 1.38
**Interruptions in care (*N* = 39)**				
No interruption in care	13	33.3	-	-
One or more interruptions < 1 month	3	7.7	-	-
One or more interruptions ≥ 1 to < 3 months	7	17.9	-	-
One or more interruptions ≥ 3 months	16	41.0	-	-
**Number of times treated for tuberculosis (*N* = 39)**				
0	19	48.7	-	-
1	17	43.6	-	-
2	3	7.7	-	-
**Number of hospitalisations pre-switch (*N* = 39)**				
0	7	17.9	-	-
1	17	43.6	-	-
2	9	23.1	-	-
> 3	6	15.4	-	-
**Number of children who never had a documented VL ≤ 1000 copies/mL**	12	30.8	-	-
**Number of children that had a resistance test**	12	30.8	-	-

ABC, abacavir; 3TC, lamivudine; LPV/r, lopinavir/ritonavir; AZT, zidovudine; d4T, stavudine; EFV, efavirenz; NVP, nevirapine; ATV/r, atazanavir/ritonavir; DRV/r, darunavir/ritonavir; TB, tuberculosis; VL, viral load; IQR, interquartile range.

**TABLE 2:** Clinical characteristics of children at the time of switching to dolutegravir and viral suppression rates (*N* = 39).

Characteristic	*n*	%	Median	IQR
**Age at switch (years) (*N* = 39)**	-	-	7.19	3.02; 9.73
< 1	0	0	-	-
≥ 1 to < 5	15	38.5	-	-
≥ 5 to < 10	15	38.5	-	-
≥ 10	9	23.1	-	-
**Hospitalisation at switch**	8	20.5	-	
**Viral load at switch (*N* = 27)**	-	-	3868	108.00; 54 550.00
**CD4+ T-lymphocyte percent and count at switch (*N* = 17)**	-	-	-	-
CD4+ T-lymphocyte percent	-	-	17.46	11.34; 22.25
CD4+ T-lymphocyte count	-	-	485	353; 746
**On TB treatment at switch**	6	15.4	-	-
**Regimen changed to at switch¶ (*N* = 39)**	-	-	-	
TDF/3TC/DTG	**6**	**15.4**	-	-
Prior AZT or d4T	5	83.3	-	-
Prior ABC†	6	100.0	-	-
ABC/3TC/DTG	**31**	**79.5**	-	-
Prior AZT or d4T	13	41.9	-	-
Prior ABC†	31	100.0	-	-
AZT/3TC/DTG	**2**	**5.1**	-	-
Prior AZT or d4T	0	0.0	-	-
Prior ABC†	2	100.0	-	-
**Viral load post-switch (*N* = 38§)**	-	-	-	-
Suppressed, VL ≤ 50 copies/mL	21	55.3	-	-
Not suppressed	**17**	**44.7**	-	-
> 50 to < 1000 copies/mL	9	52.9	-	-
≥ 1000 copies/mL	8	47.1	-	-
**Time from switch to time of viral load used to determine not suppressed (months) (*N* = 17)**	-	-	9.33	4.76; 19.32
< 6	7	41.2	-	-
≥ 6 to < 12	0	0.0	-	-
≥ 12	10	58.8	-	-
**Time from switch to time of viral load used to determine suppressed (months) (*N* = 21)**	-	-	11.03	4.30; 22.30
< 6	8	38.1	-	-
≥ 6 to < 12	3	14.3	-	-
≥ 12	10	47.6	-	-
**Interruptions in care (*N* = 39)**	-	-	-	-
No interruption in care	24	61.5	-	-
One or more interruptions < 1 month	7	17.9	-	-
One or more interruptions ≥ 1 to < 3 months	6	15.4	-	-
One or more interruptions ≥ 3 months	2	5.1	-	-
**TB diagnosed post-switch**	2‡	5.1	-	-
**Number of hospitalisations post-switch (*N* = 39)**	-	-	-	-
0	31	79.5	-	-
1	3	7.7	-	-
> 2	5	12.8	-	-
**Total duration on dolutegravir at our clinic (months) (*N* = 39)**	-	-	9.23	5.88; 25.79
< 6	10	25.6	-	-
≥ 6 to < 12	11	28.2	-	-
≥ 12	18	46.2	-	-
**Anthropometry at switch**				
Weight (kg) (*N* = 36)	-	-	19.15	12.16; 33.19
< 20 kg	20	51.3	-	-
≥ 20 to < 30 kg	7	19.4	-	-
≥ 30 kg	9	25.0	-	-
Weight (*Z* score)	36	-	−1.145	−1.84; −0.11
Height (cm)	33	-	116	94; 137
Height (*Z* score)	33	-	−1.36	−2.20; −0.62
BMI (*Z* score)	33	-	−0.04	−0.71; 0.96
Time from date of switch to date of last clinic visit anthropometry (years)	39	-	0.77	0.41; 2.15
**Anthropometry at last clinic visit**				
Weight (kg)	39	-	21.00	13.60; 45.60
Weight (*Z* score)	39	-	−0.56	−1.48; 0.06
Height (cm)	31	-	121	106; 155
Height (*Z* score)	31	-	−1.25	−2.01; −0.56
BMI (*Z* score)	31	-	0.37	−0.29; 1.17

TDF, Tenofovir disoproxil fumarate; 3TC, lamivudine; DTG, dolutegravir; AZT, zidovudine; d4T, stavudine; ABC, abacavir; TB, tuberculosis; BMI, body mass index; IQR, interquartile range.

† , All children were exposed to ABC pre-switch.

‡ , One child was treated at switch but had an additional episode of treatment post-switch, likely because of defaulting; one child was not treated with rifampicin-based treatment.

§ , A recorded post-switch viral load was available for 38 children. One child, aged 3 years, had been on ABC/3TC/DTG for 6 months but had no repeat viral load at study end.

¶ , Bold figures represent the total number and percentage of children in each regimen category. Indented rows show prior nucleoside reverse transcriptase inhibitor exposure.

Notably, eight children (20.5%) were hospitalised at the time of the switch, and six children (15.4%) were on rifampicin-based anti-tuberculosis therapy (Table 1).

Prior to switching to dolutegravir, all children were receiving abacavir and lamivudine. Nearly all of the children (*n* = 38, 97.4%) had received one or more protease inhibitors (Table 1). Twelve children (30.8%) had never achieved a documented viral load of ≤ 1000 copies/mL prior to initiating dolutegravir (Table 1). Interruptions in care were common, with 23 children (59.0%) experiencing at least one interruption in treatment exceeding 1 month, prior to the switch (Table 1).

Only six children (15.4%) were switched to TDF/lamivudine/dolutegravir, and no children received zidovudine (Table 2). Among the 21 children (55.3%) who achieved viral suppression by the end of the study, eight (38.1%) had been on therapy for < 6 months (Table 2). Of the 17 children (44.7%) with persistent viral non-suppression (Table 2), seven (41.2%) had a viral load between 50 and < 400 copies/mL. Six of the seven children (85.7%) on treatment ≤ 6 months had low-level viraemia (50–400 copies/mL).

Abacavir was recycled in 31 children (available viral load for 30/31), of whom 50.0% achieved viral suppression (Table 2 and Table 3). The difference in suppression rates between children receiving a TDF-containing backbone and an abacavir-containing backbone was not statistically significant. However, the number of children receiving a TDF-containing backbone was small, limiting meaningful comparison. Duration of treatment prior to switch, age at treatment initiation, age at switch to dolutegravir, and availability of resistance testing before switch were not predictive of persistent viral non-suppression (Table 3). Fifteen children (38.5%) experienced documented interruptions in care after switching to dolutegravir. Of these, 12 (80.0%) had also interrupted therapy at least once prior to the switch.

**TABLE 3:** Risk factors for viral non-suppression after switching to dolutegravir.

Characteristic	Suppressed (*N* = 21)				Not suppressed (*N* = 17)				*P*
	*n*	%	IQR	Median	*n*	%	IQR	Median	
**Sex**									
Female	12	57.1	-	-	6	35.3	-	-	0.21
**Age at treatment initiation (years)**	-	-	0.18; 1.53	0.47	-	-	0.28; 2.02	1.13	0.87
< 1	12	57.1	-	-	8	47.1	-	-	-
≥ 1 to < 5	8	38.1	-	-	8	47.1	-	-	-
≥ 5 to < 18	1	4.8	-	-	1	4.8	-	-	-
**Age at switch to DTG (years)**	-	-	3.91; 12.70	7.50	-	-	1.90; 9.62	5.07	0.25
< 1	0	0.0	-	-	0	0.0	-	-	-
≥ 1 to < 5	6	28.6	-	-	8	47.1	-	-	-
≥ 5 to < 10	8	38.1	-	-	7	41.2	-	-	-
≥ 10	7	33.3	-	-	2	11.8	-	-	-
**Duration on treatment pre-switch (years)**	-	-	2.15; 11.34	6.86	-	-	1.09; 7.32	4.98	0.63
< 2	5	23.8	-	-	6	35.3	-	-	-
≥ 2 to < 4	3	14.3	-	-	1	5.9	-	-	-
≥ 4	13	58.8	-	-	10	58.8	-	-	-
**ART exposure pre-switch**									
ABC/3TC/LPV/r	8	38.1	-	-	11	64.7	-	-	-
ABC/3TC/LPV/r / (AZT or D4T)	4	19.1	-	-	1	5.9	-	-	-
ABC/3TC/LPV/r/ (AZT or D4T)/ (EFV or NVP)	4	19.1	-	-	3	17.7	-	-	-
ABC/3TC/ (EFV or NVP)	1	4.8	-	-	0	0.0	-	-	-
ABC/3TC/ (LPV/r and ATV) / (AZT or D4T)	4	19.1	-	-	1	5.9	-	-	-
ABC/3TC/LPV/r/ (AZT or D4T)/ (EFV or NVP)/DRV/r	0	0.0	-	-	1	5.9	-	-	-
**Resistance test performed prior to switch**	5	22.7	-	-	7	43.8	-	-	0.29
**NRTI combined with 3TC and DTG†**									
TDF	**5**	**23.8**	-	-	1	5.9	-	-	0.41
Prior AZT or d4T	4	80.0	-	-	1	100.0	-	-	-
Prior ABC	5	100.0	-	-	1	100.0	-	-	-
ABC	**15**	**71.4**	-	-	15	88.2	-	-	-
Prior AZT or d4T	8	53.3	-	-	5	33.3	-	-	-
Prior ABC‡	15	100.0	-	-	15	100.0	-	-	-
AZT	**1**	**4.8**	-	-	1	5.9	-	-	-
Prior AZT	0	0.0	-	-	0	0.0	-	-	-
Prior ABC	1	100.0	-	-	1	100.0	-	-	-
**Interruptions in care pre-switch**									
No interruption in care	6	28.6	-	-	7	41.2	-	-	0.50
Interruptions in care	15	71.4	-	-	10	58.8	-	-	-
**Interruptions in care post-switch**									
No interruption in care	16	76.2	-	-	8	47.1	-	-	0.09
Interruptions in care	5	23.8	-	-	9	52.9	-	-	-
**Hospitalisation at switch**	4	18.2	-	-	4	25.0	-	-	0.70
**On TB treatment at switch**	2	9.1	-	-	4	25.0	-	-	0.22

IQR, interquartile range; DTG, dolutegravir; ART, antiretroviral therapy; ABC, abacavir; 3TC, lamivudine; LPV/r, lopinavir/ritonavir; ATV/r, atazanavir/ritonavir; AZT, zidovudine; d4T, stavudine; EFV, efavirenz; NVP, nevirapine; DRV/r, darunavir/ritonavir; NRTI, nucleos(t)ide reverse transcriptase inhibitor; TB, tuberculosis.

† , Bold figures represent the total number and percentage of children switched to each NRTI-containing regimen (combined with 3TC and DTG). Indented rows show prior nucleoside reverse transcriptase inhibitor exposure.

‡ , All children were exposed to ABC pre-switch.

Twelve children (30.8%) had one or more resistance tests (Table 1). Only one child had resistance testing performed after switching to dolutegravir (Table 4). Of the eight children receiving dolutegravir with an abacavir-containing backbone who had a resistance test result available, five (66.7%) had some documented resistance to abacavir. Four of those five children suppressed.

**TABLE 4:** Drug exposure, resistance mutations & viral load outcomes of 12 children with available data.

PID	Time on ART at switch (years)	Drugs Exposed	Resistance test		NRTI scores				NNRTI scores			PI scores			NRTI post	Last VL captured (copies/mL)
			Drug class	Mutations	ABC	AZT	TDF	3TC	EFV	RPV	ETR	ATV	LPV	DRV		
3	6.5	ABC/3TC-LPV	NRTI	M184V	15	−10	−10	60	-	-	-	-	-	-	ABC	≤ 50
			NNRTI	None												
			PI	None												
7	7.5	ABC/3TC-LPV	NRTI	None	-	-	-	-	0	15	10	-	-	-	ABC	> 1000
			NNRTI	E138A												
			PI	None												
11	4.5	ABC/3TC-LPV	NRTI	M184V	15	−10	−10	60	-	-	-	-	-	-	ABC	≤ 50
			NNRTI	None												
			PI	None												
12	1.6	ABC/3TC-LPV	NRTI	M184V; L74V; V75M	60	0	−10	60	105	0	0	40	55	0	AZT	> 50 – ≤ 1000
			NNRTI	K103N; P225H; V179I/V												
			PI	I54V; V82A												
20	13.1	ABC/3TC/d4T-LPV	NRTI	None	-	-	-	-	60	0	0	-	-	-	ABC	≤ 50
			NNRTI	V106M												
			PI	None												
22	0.9	ABC/3TC-LPV	NRTI	M184V; L74V	60	−10	−10	60	30	45	30	-	-	-	ABC	> 50 – ≤ 1000
			NNRTI	Y181C												
			PI	None												
25¶	15.2	ABC/3TC/d4T-LPV/ATV	NRTI	M184V	15	−10	−10	60	-	-	-	-	-	-	TDF	≤ 50
			NNRTI	None												
			PI	None												
26†	14.0	ABC/3TC/d4T-LPV	NRTI	M184V	15	−10	−10	60	-	-	-	-	-	-	TDF	> 1000
			NNRTI	None												
			PI	None												
27†	3.0	ABC/3TC/AZT-LPV-NVP	NRTI	None	-	-	-	-	-	-	-	-	-	-	ABC	> 50 – ≤ 1000
			NNRTI	None												
			PI	None												
28¶	5.0	ABC/3TC/AZT-LPV/DRV-NVP	NRTI	M184V; L74V; L74L/V	60	−10	−10	60	30	45	30	80	140	45	ABC	> 1000
			NNRTI	Y181C; K101K/Q												
			PI	I54V; L76V; M46I; V82A; L10F; V32VI												
30†	1.0	ABC/3TC-LPV	NRTI	M184V; L74V; Y115F	105	−10	5	60	60	0	0	65	120	25	AZT	≤ 50
			NNRTI	K103N												
			PI	I54V; L76V; M46I; V82A; L33F												
36	6.4	ABC/3TC-LPV	NRTI	M184V	15	−10	−10	60	-	-	-	-	-	-	ABC	> 50 – ≤ 1000
			NNRTI	None												

PID, patient identifier; NRTI, nucleos(t)ide reverse transcriptase inhibitor; NNRTI, non-nucleoside reverse transcriptase inhibitor; PI, protease inhibitor; VL, viral load; DTG, dolutegravir; ABC, abacavir; 3TC, lamivudine; LPV, lopinavir; ATV/r, atazanavir/ritonavir; AZT, zidovudine; d4T, stavudine; EFV, efavirenz; NVP, nevirapine; DRV, darunavir; TDF, Tenofovir disoproxil fumarate; RPV, rilpivirine; ETR, etravirine; ART, antiretroviral therapy.

† , Resistance test prior to ART exposure and pre-switch.

‡ , Only child to have a resistance test after switching to a dolutegravir-based regimen. M184V mutation present pre-switch. No integrase resistance or M184V mutation present following 28 months on TDF/3TC/DTG, suggesting non-adherence to treatment. This was the only child on TDF that did not suppress.

¶ , More than one resistance test pre-switch.

**FIGURE 1:**
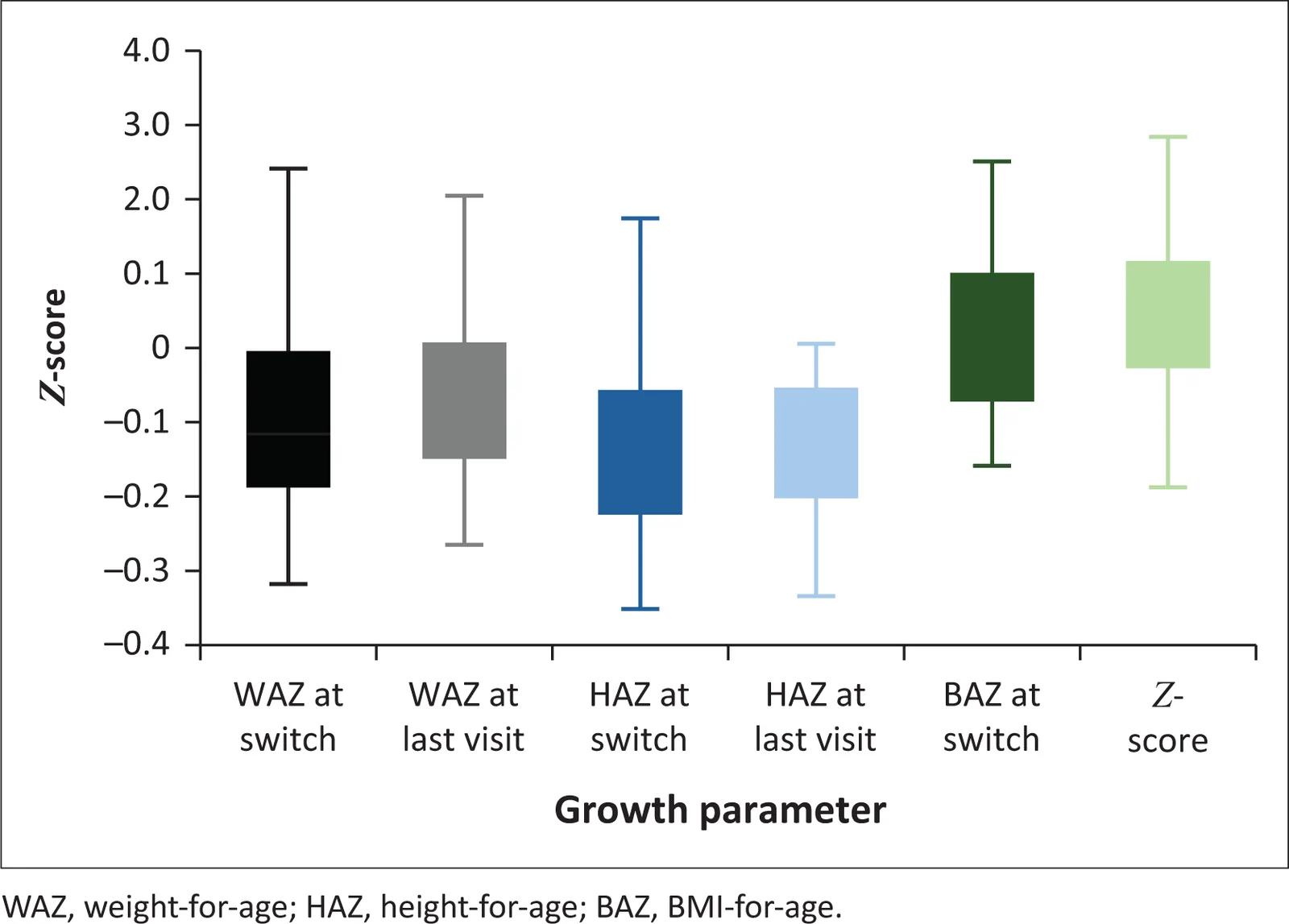
Weight-for-age, height-for-age, and body mass index-for-age *Z*-scores at switch and at last visit.

After switching to a dolutegravir-based regimen, eight children (21.5%) required hospitalisation (Table 2). We did not review the indications for hospitalisation. Two children (5.1%) developed tuberculosis, including one case of rifampicin-resistant disease (Table 2). Among children with available data, the median BMI *Z*-score at the last documented clinic visit was 0.37 (IQR –0.29; 1.17), representing an increase from a median BMI *Z*-score of –0.04 (IQR –0.71; 0.96) at the time of dolutegravir initiation (Figure 1 and Table 2). Dolutegravir was not discontinued in any child because of an adverse event.

## Discussion

We present South African programmatic data on the virological outcomes of children switched to a dolutegravir-based regimen while experiencing persistent viral non-suppression. At median follow-up of 11.03 months (IQR 3.30; 22.30), 55.3% of children achieved viral suppression to ≤ 50 copies/mL. However, a significant number of children had only a single follow-up viral load measurement, often obtained less than 6 months after switching to dolutegravir.

It appears that the outcomes in our cohort are less favourable than those reported in the ODYSSEY and CHAPAS-4 trials.^12,16^ In CHAPAS-4, at 48 weeks, more than 90% of children achieved viral suppression to < 400 copies/mL (primary outcome). The study also showed that more than 80% of children achieved suppression to < 60 copies/mL from approximately 6 weeks after initiation of dolutegravir, which was sustained throughout the main study period and extended follow-up.^16^ Similarly, in ODYSSEY Part B, among the 196 children switched to dolutegravir while experiencing persistent viral non-suppression, 81% (95% CI 75–86) achieved viral loads < 50 copies/mL and 89% (95% CI 8 4–93) achieved viral loads < 400 copies/mL at 96 weeks.^12^ This highlights important differences between clinical trial populations and real-world programmatic cohorts. Our cohort consisted of younger children compared with those enrolled in CHAPAS-4 and ODYSSEY Part B.^12,16^ At dolutegravir initiation, the median age in our cohort was 7.19 years (IQR 3.02; 9.73), with 15 children (38.5%) aged < 5 years and only nine (23.1%) aged ≥ 10 years. In contrast, CHAPAS-4 reported a median age of 11 years (IQR 8; 13), with only six children (2.6%) aged 3–4 years and the majority (139, 60.7%) aged 10–15 years. Similarly, in ODYSSEY (Parts A and B) the median age was 12.2 years (IQR 9.2; 15.1). These age differences were also reflected in weight band distributions. The median weight in CHAPAS-4 was 27 kg (IQR 21.3; 34.0), and in ODYSSEY (Parts A and B), 30.4 kg (IQR 23.7; 43.7).^12,16^ In contrast, 20 children (51.28%) in our cohort weighed < 20 kg, while only nine children (25.0%) weighed ≥ 30 kg. This limited available therapeutic options, as lower weights precluded the use of the once-daily FDC tablets available in the South African HIV programme at the time. The recent introduction of a paediatric dispersible abacavir/lamivudine/dolutegravir FDC (pALD), together with the availability of generic dolutegravir-based FDCs with a TAF-containing backbone, is expected to improve this situation. Although pALD is dispersible, children using pALD still need to take three to six tablets per day.^32^

Although our cohort was relatively young, the number of children was insufficient to draw firm conclusions regarding outcomes in those weighing < 14 kg. In ODYSSEY Part B, 13 children weighed < 14 kg, of whom seven were assigned to the dolutegravir arm.^14^ Across ODYSSEY Parts A and B, treatment failure at 96 weeks occurred in 31% of children weighing < 14 kg on dolutegravir, compared with 13.4% among those weighing ≥ 14 kg.^12,14^ This is consistent with existing evidence that infants and younger children take longer to achieve viral suppression and are possibly at higher risk of persistent viral non-suppression.

In ODYSSEY Parts A and B, there is no statistically significant difference in weight gain between dolutegravir and standard-of-care groups at follow-up. The mean differences in weight (dolutegravir minus standard of care) were 1.0 kg (95% CI –0.2 to 2.2; *P* = 0.0950) in the ≥ 14 kg cohort at 240 weeks’ follow up, and –0.1 kg (95% CI –0.8 to 0.7; *P* = 0.83) in the < 14 kg cohort at 192 weeks’ follow up.^33^ We present short-term weight outcomes, with the median weight-for-age *Z*-scores increasing from –1.145 (–1.84; –0.11) at switch to –0.56 (–1.48; 0.06), suggesting a return to health.

Although resistance to dolutegravir is rare in children initiating dolutegravir-based first-line therapy, it occurs more frequently among those who switch to dolutegravir in the context of viral failure.^23^ In both CHAPAS-4 and ODYSSEY Part B, treatment failure was not primarily driven by dolutegravir resistance.^12,16^ In CHAPAS-4, two of nine (22%) children receiving dolutegravir who experienced persistent viral non-suppression and underwent resistance testing had evidence of dolutegravir resistance. Notably, both had received zidovudine as part of their ART regimen.^34^ Similarly low rates of resistance were reported in ODYSSEY Part B, where 18% (4/22) of children with persistent viral non-suppression demonstrated resistance to integrase strand transfer inhibitors.^12^ We were unable to assess the development of dolutegravir resistance in our cohort, as only one child underwent resistance testing following the switch to dolutegravir. This child had the M184V mutation prior to switch but, after 28 months of treatment with TDF, lamivudine and dolutegravir, repeat resistance testing did not demonstrate integrase resistance or a detectable M184V mutation, suggesting non-adherence. This was the only child receiving TDF that experienced persistent viral non-suppression. A key distinction between our cohort and those enrolled in clinical trials is prior ART exposure. Most children in our cohort were protease inhibitor experienced, whereas participants in CHAPAS-4 and ODYSSEY Part B largely had ongoing viraemia while receiving non-nucleoside reverse transcriptase inhibitor-based regimens.^16,35^ While this difference is unlikely to influence the outcomes of dolutegravir-based regimens, it may have implications for further management if protease inhibitor resistance went undetected.

In CHAPAS-4, children receiving TAF achieved higher rates of viral suppression than those receiving abacavir or zidovudine,^16^ findings consistent with adult data supporting the reuse of TDF over zidovudine. However, TAF was not available for paediatric use locally, and very few children in our cohort met weight thresholds for TDF. Although the proportion of children achieving viral suppression on TDF (5/6) was higher than among those receiving abacavir (15/30), the numbers are small and this difference did not reach statistical significance. However, in light of the CHAPAS-4 study results, the urgent need to improve access to TAF to optimise treatment outcomes for children switching to new ART regimens in order has become evident.

### Limitations

This study had several limitations. Only routinely collected data from secondary data sources were available, resulting in missing data. Additionally, the small sample size limits generalisability to the broader population of CALHIV in the province or South Africa.

The observed discrepancies between trial data and programmatic data highlight the importance of evaluating outcomes following guideline implementation in real-world settings. Furthermore, we observed high rates of treatment interruption both prior to and, importantly, following the switch to a dolutegravir-based regimen. Timely clinic attendance and regular pharmacy collection have been shown to correlate with viral suppression in adults.^36^ In this study we did not assess regimen tolerability, acceptability or social stressors as additional factors that may influence regular clinic attendance and viral suppression rates. Delayed clinic attendance and missed pharmacy collections may raise concerns for poor adherence, particularly in this group of children with a history of significant treatment interruptions.^36^ Supporting families to maintain adherence and ensure consistent clinic attendance is essential and requires attention and dedicated resources.

Additionally, this work highlights the need to clearly understand the adherence profiles and drug combinations that predispose patients to dolutegravir resistance. Given that poor engagement with care remains the strongest predictor of treatment failure, it is essential to define when and for whom resistance testing should be prioritised. A cost–benefit analysis evaluating early resistance testing after 1 year of persistent viraemia on second-line dolutegravir-based regimens is required to guide more targeted and effective use of limited resources.^37^

## Conclusion

Viral suppression in this real-world cohort was lower than that reported in clinical trials. However, given the small sample size and observational study design, generalisabilty of the data is limited. That viral suppression remained suboptimal despite switching to simpler and more tolerable regimens suggests that regimen optimisation must be supported by social interventions to further support adherence.

Our findings highlight the potential importance of more comprehensive models of care, including community-based, financial and psychosocial support to better support CAHIV and their families.
